# Supplementary material for: Staggered structural dynamic-mediated selective adsorption of H2O/D2O on flexible graphene oxide nanosheets
Source: Nat Commun. 2024 Apr 27;15:3585. doi: 10.1038/s41467-024-47838-9 (PMC11055881; doi:10.1038/s41467-024-47838-9)
Supplement: Supplementary file 1 — Supplementary Information [file 41467_2024_47838_MOESM1_ESM.pdf]

Staggered structural dynamic-mediated selective adsorption of H_2O/D_2O on flexible graphene oxide nanosheets

Ryusuke Futamura[1,2], Taku Iiyama[1,2], Takahiro Ueda[3], Patrick A. Bonnaud[4], François-Xavier Coudert[5], Ayumi Furuse[2], Hideki Tanaka[2], Roland J. -M. Pellenq[6] and Katsumi Kaneko[2]*

[1]Faculty of Science, Department of Chemistry, Shinshu University, 3-1-1, Asahi, Matsumoto, 390-8621, Japan

[2]Research Initiative for Supra-Materials, Shinshu University, 4-17-1, Wakasato, Nagano, 380-8553, Japan

[3]Faculty of Science, Osaka University, 1-13, Machikaneyamacho, Toyonaka, 560-0043, Japan

[4] Institute for Materials Research, Tohoku University, Katahira 2-1-1, Aoba, Sendai 980-8577, Japan

[5] Chimie Paris Tech, PSL University, CNRS, Institut de Recherche de Chimie Paris, 11 Pierre and Marie Curie 75231, Paris, France

[6] European Institute of Membranes (IEM), CNRS and the University of Montpellier, 300 Avenue du Professeur Jeanbrau, 34090 Montpellier, France

*e-mail: kkaneko@shinshu-u.ac.jp

Supplementary information

Content

1. Note on modeling isotope quantum effect in D_2O versus H_2O

2. H_2O selective adsorption on GO from the vapor of H_2O/D_2O mixture

3. Supplemental figures and tables

1. Note on modeling isotope quantum effect in D_2O versus H_2O

Quantum effects in water should be investigated by implementing the Feynman–Hibbs approach (QFH) using a reference potential for H_2O and D_2O published in B. Guillot and Y. Guissani. *J. Chem Phys.* 108(24), p 22, 1998, "Quantum effects in simulated water by the Feynman–Hibbs approach". In this work, the evolution of the thermodynamics, the structure, the diffusivity, and the dynamics in light and heavy water is investigated over a large range of temperature and is compared with experimental data and with classical simulations as well. The accuracy of the results and the very low cost in computer time make the Feynman–Hibbs approach a valuable procedure to rapidly estimate the order of magnitude of the quantum contributions to intermolecular properties of water.

From the path-integral quantum partition function (without exchange) for a canonical ensemble (*N*, *V*, *T*) of atoms, and after some algebra, the FH potentials can be obtained. By keeping quadratic fluctuations around the classical path, one obtains the QFH potential:

$$U_{QFH}(r) = U_C(r) + \frac{\beta\hbar^2}{24\mu}\left[U_C^{''}(r) + 2\frac{U_C^{'}(r)}{r}\right] \tag{1}$$

This potential is built so as to improve upon a classical interaction model C, normally Lennard-Jones (LJ) + Coulombic for H_2O and D_2O, by taking into account factors related to quantum features (with $\hbar=h/2\pi$, h the Plank constant, the effective mass $\mu = m_1.m_2/m_1+m_2$, temperature $\beta = 1/kT$). The estimate of the quantum effects by the QFH potential is only valid when the quantum corrections to classical quantities remain small. The order of magnitude of these corrections is given by the value of the parameter ($2\beta\hbar^2/m\sigma^2$) where σ can be taken equal to the De Broglie wave length ($l=h/(2\pi mkT)^{1/2}$) that is a typical length associated to the size of system molecules or atoms, for instance, equal to the σ parameter of the LJ potential modelling the interactions. For rare gas such as Ne, It is worthwhile to point out that at distances $r \leq r_m$ (LJ-minimum) the repulsive character of the QFH potential predicts a larger energy (a more positive repulsion at short distance) than the uncorrected potentials; this difference being more pronounced as *r* and *T* both decrease showing that the QFH potential tends to increase the apparent molecular size with some obvious implications regarding thermodynamics properties [cite N. Tchouar, M. Benyettou and S. Benyettou, Feynman-Hibbs Quantum Effective Potentials For Molecular Dynamic Simulations of Liquid Neon,

https://www.mdpi.org/fis2005/F.62.paper.pdf]. It is found that quantum effects are significant near ambient conditions and vanish with increasing temperature less drastically than generally assumed. The most affected quantity is the self-diffusion coefficient.

In the context of water adsorption in carbon nanoporous materials, we used the interatomic potential forms to describe water-water interaction and the isotope deuterium effect following Guillot *et al.* reparametrizing the original Rahman-Stillinger central force water potential [cite H. L. Lemberg and F. H. Stillinger, *J. Chem. Phys.* 62, 1677, 1975; A. Rahman, F. H. Stillinger, and H. L. Lemberg, ibid. 63, 5223, 1975; F. H. Stillinger and A. Rahman, ibid. 68, 666, 1978. The interatomic forms for the water bonded (described by a Morse function) and non-bonded interactions (combining an electrostatic charge-charge $1/r$ form and dispersion interactions) are given by the following equations:

$$\mathscr{V}_{\mathrm{OO}}(r)=\frac{144.358}{r}+0.5\left[\left(\frac{3.74}{r}\right)^{8}-\left(\frac{3.74}{r}\right)^{6}\right],$$

$$\mathscr{V}_{\mathrm{OH}}(r)=-\frac{72.269}{r}-\frac{4.3}{1+e^{0.9(r-2.2)}}+3.9[(e^{-5.8(r-1.07)}-1)^{2}-1],$$

$$\mathscr{V}_{\mathrm{HH}}(r)=\frac{36.1345}{r}+\frac{17}{1+e^{3.1(r-2.05)}}+13[(e^{-6.0(r-1.495)}-1)^{2}-1]. \qquad \text{(2a,2b,2c)}$$

We then calculated the quantum Hibbs Feynman correction regarding the H/D isotope effect for the last two interatomic potential functions (eq. 2b and 2c) using equation 1 that requires evaluating the analytical first and second derivatives with respect to distance (evaluated using Chat-GTP and Wolfram-Alpha web applications) and the $\beta\hbar^2/24m$ factor. At room temperature, its values for the H-H, D-D, H-O and D-O pairs are $1.94.10^{-2}$, $9.70.10^{-3}$, $1.02.10^{-2}$, $5.51\ 10^{-3}$ kcal/mol respectively using the molar mass of O, H and D at 16, 1 and 2 g/mol. Figure S1 presents the change in the various interatomic potentials involving H(D) pairs at 300 K.

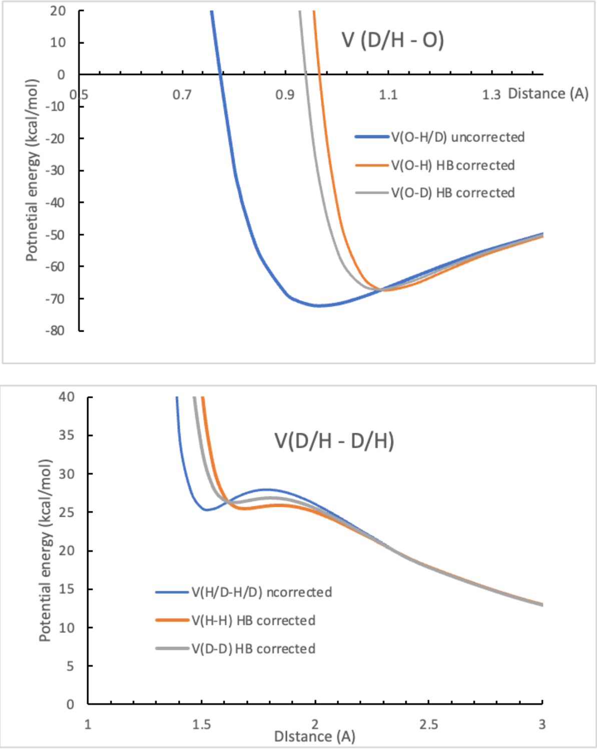

Figure S1| Hydrogen bond corrections regarding the isotopic effect on bonded and non-bonded interatomic potentials at 300 K calculated with the Hibbs-Feynman approach (see Eq. 1) and using the Rahman-Stillinger water model (see text).

One can see from Figure S1 that the isotope effect at room temperature leads to a globally softer and smaller D_2O molecules as compared to the pristine H_2O: the bonded O – D distance is actually shorter by 2.32% than the O – H one and the short-range repulsive wall is actually less stiff for the O – D pair as compared with the O – H one. We note the same behavior for the D – D and H – H pairs where the D – D short-range repulsion is actually softer than that for the H – H pair while the D – D is 2.35% shorter than the H – H distance at the potential minimum (that is the equilibrium distance between 2 H's / 2 D's within a given H_2O / D_2O molecule). Calculating the D-O-D angle satisfying to both the D-O bond length and the D-D distance within a D_2O molecule gives a D-O-D angle at 99.75, *i.e.* smaller than the H-O-H angle. Interestingly enough, the dipole moment (calculated from the molecule mass center that is located on the *z*-axis going through the O location and the mid-point of the D-D distance at $z = 0.1399$ Å taking the O location as frame reference) turns out to be slightly larger ($\mu = 1.865$D) to that of the pristine H_2O molecule ($\mu = 1.850$D). This leads to a denser packing of D_2O molecules in dense liquid/solid phases (a globally smaller molecule with slightly stronger dipole-dipole interactions) leading to a more structured D_2O liquid with a larger heat of

vaporization compared to liquid H_2O in agreement with experiment [cite CRC Handbook of Chemistry and Physics: A Ready-Reference Book of Chemical and Physical Data. 2003–2004, 84th ed., edited by D. R. Lide (CRC, Boca Raton, FL; London, 2003)]. Our results agree well with the experimental data on liquid H_2O and D_2O of Soper and Benmore [cite A. K. Soper, C. J. Benmore, Quantum Differences between Heavy and Light Water, Physical Review Letters, 101, 065502 (2008)] who found that heavy water is a more structured liquid than light water with in particular the O – H bond length in H_2O 3% longer than the O – D bond length in D_2O. The intermolecular H – H distance is 2% longer in H_2O compared to that in D_2O. This reflects in the higher average number of hydrogen bonds per molecule in D_2O (3.76) than in H_2O (3.62) [cite Timothy Clark, Julian Heske, and Thomas D. Kühne, Opposing Electronic and Nuclear Quantum Effects on Hydrogen Bonds in H_2O and D_2O, ChemPhysChem 2019, 20, 2461-2465].

In a confined situation as in GO, this will lead to molecular packing driven by the D_2O-OH groups and D_2O-D_2O interactions. Confinement frustrates the D-bond network formation. The fact that the interaction between D_2O-D_2O is larger (than that for H_2O) will lead to a peculiar situation that for a given OH groups concentration, GO is more D_2O-phobic than H_2O-phobic (acknowledging the fact that increasing OH surface group density will switch from a totally hydrophobic substrate (only able to accommodate water in its pores from the pressurized liquid outside) to a more philic situation *i.e* allowing water intake from a vapor phase containing outside reservoir (i.e at $P/P_0 = R_h$ less than 100%, typically 50%)) at room temperature. It is therefore expected that isotope effects will lead to a lower adsorbed quantities for D_2O compared to that for H_2O at a given relative humidity and temperature supporting experimental findings reported in this work.

2. H_2O selective adsorption on GO from the vapor of H_2O/D_2O mixture

Using mass spectroscopy, we conducted adsorption measurements of the mixed vapor of H_2O and D_2O on GO to confirm the hydrogen isotopic water selective adsorption characteristics of GO. Figure S2(a) shows a schematic of the lab-made vapor adsorption line equipped with a quadrupole mass spectrometer (M-101QA-TDF, Canon ANELVA Co.) and a turbomolecular vacuum pump (T-Station 75, Edwards Co.). A GO sample (0.221 g) was used to determine the composition of the desorbed water mixture. Water obtained by filtration of distilled water with a Milli-Q Reference water system (Millipore Japan Co.) was used as H_2O. Here, the content of deuterium in H_2O was negligible (143 ± 2 ppm, Y. Ono, R. Futamura, K. Kaneko et al., *J. Colloid. Interface Sci.* 508 (2017) 14–17). Deuterium oxide (99.8 atom% deuterated, Kanto Chemical Co., Inc.) was used as D_2O. GO was pretreated at 333 K under vacuum (< 0.1 Pa) for 2 h prior to the adsorption measurements. We prepared an H_2O/D_2O liquid mixture with a mole fraction of H_2O:D_2O = 1.13:1.00 (i.e., H/D = 1.13) in a liquid reservoir of the adsorption line. Herein, we describe the composition of the mixture using the H/D ratio, as the formation of HDO molecules inevitably occurs in the H_2O/D_2O mixture. We refer to the hydrogen isotopic water mixture as an "H_2O/D_2O mixture," even though the mixture contains HDO whose composition varies with the mixture composition of H_2O and D_2O at a constant temperature.

The 15 g of liquid mixture that was used in this experiment for the mixed feed vapor was sufficiently large compared to the amount adsorbed (i.e., ~90 mg). Thus, the change in the liquid H_2O/D_2O composition due to adsorption was negligibly small. The liquid mixture in the liquid reservoir was frozen at 77 K with liquid N_2 prior to mixed-vapor adsorption and purified by removing the dissolved air through evacuation under melting conditions three times. The mixed-vapor adsorption of H_2O and D_2O was conducted near the saturation vapor pressure because the difference in the amount of adsorption between H_2O and D_2O in the single-component adsorption isotherms near the saturation vapor pressure (Fig. 4(a)) was sufficiently large for reliable measurement of the compositional change of H_2O and D_2O in the mixed-vapor adsorption. The temperature of the H_2O/D_2O liquid mixture in the liquid reservoir was kept constant at 297 ± 0.2 K, and the vapor was adsorbed on GO at 298 K for 1 d to achieve adsorption equilibrium at a relative vapor pressure of 0.94. Here, the relative vapor pressure for pure H_2O liquid was used because the derivation of the relative vapor pressure of the H_2O/D_2O mixture was difficult due to H/D exchange.

The adsorbed H_2O and D_2O molecules were completely desorbed from the GO at 333 K following adsorption equilibration, and the desorbed H_2O and D_2O vapors were collected

in a cold trap at 77 K for 2 h. We confirmed the entire desorption of H_2O and D_2O from GO using weight loss measurements of GO adsorbing water molecules under the desorption conditions of 333 K in vacuum (< 0.1 Pa) for 2 h (Table S4).

The adsorption selectivity of GO for H_2O and D_2O was determined by measuring I^{18} and I^{20}, which are the mass intensities at $m/z = 18$ and 20, respectively. Because the observed compositions of H_2O and D_2O can provide the HDO content when the equilibrium relation is used, we only show the results for H_2O and D_2O.

The H/D ratio of the H_2O/D_2O mixture and the mass intensities were calibrated in advance (Fig. S13). As shown in Fig. S2(b), we measured the time courses of the mass spectra of the feed vapor and the vapor of collected H_2O/D_2O mixture adsorbed on GO due to the faster evaporation rate of lighter isotopes of H_2O in the early stages of the measurements. Then, we obtained reliable I^{18}/I^{20} values of the equilibrated composition of the vapors by averaging the curves over 100 s from 200 to 300 s. The preferential adsorption of H_2O on GO over that of D_2O was confirmed by the higher mass intensity ratio of I^{18}/I^{20} for the desorbed vapor ($I^{18}/I^{20} = 2.40$) than that for the feed vapor ($I^{18}/I^{20} = 1.25$). The H/D ratio of the H_2O/D_2O mixture was determined from a calibration curve of the mass spectral intensity ratio of I^{18}/I^{20} versus the H/D ratio of the H_2O/D_2O mixture (Fig. S13). The corresponding H/D ratios of the feed vapor and desorbed vapor after adsorption on GO were 1.13 and 1.70, respectively. This indicated a higher H content in the adsorbed mixture on GO than in the feed vapor (i.e., the amount of H_2O is greater than that of D_2O in the adsorbed mixture).

Table S1 compares the amounts of H_2O and D_2O adsorbed on GO for single-component and mixed-vapor adsorption. Here, an unclear comparison between single-component and mixed-vapor adsorption should be avoided due to the predominantly formed HDO in the H_2O/D_2O mixture, as chemical exchange occurs based on the following equilibrium constant:

$$K = \frac{[\mathrm{HDO}]^2}{[\mathrm{H_2O}][\mathrm{D_2O}]}$$
$$= 3.85 \text{ (at 298 K)}$$

Therefore, we determined the H/D ratio of hydrogen isotopic water mixtures using mass intensity measurements and compared it with the H/D value obtained from single-component adsorption measurements. The adsorption amount of the hydrogen isotopic water mixture on GO was 399 mg g^{-1}, as determined by the weight measurement of GO following the mixed H_2O/D_2O vapor adsorption. The corresponding adsorption amounts of H_2O, D_2O, and HDO in the mixed-vapor adsorption were 8.36, 2.91, and 9.68 mmol/g, respectively. Here, H/D =1.70 and the equilibrium constant (K = 3.85) for the isotopic

exchange reaction were used to determine the adsorption amounts for each component. The adsorption amounts for single-component adsorption at P/P_0 = 0.94 were 14.1 and 10.9 mmol g^{-1} for H_2O and D_2O, respectively, which were scaled by 0.5 to compare with those of the 1:1 mixed-vapor adsorption. The corresponding H/D ratio of single-component adsorption was 1.29, and the higher H/D ratio under mixed-vapor adsorption (i.e., H/D =1.70). This indicates that the D_2O-phobicity of GO was promoted because of selective H_2O adsorption even on the unfavorable adsorption sites of the oxygen functional groups for D_2O as compared with that of single-component adsorption.

Saidi et al. reported that D_2O can be strongly adsorbed on GO, and D_2O desorption from GO is more difficult with their theoretical calculation for water molecules on GO sheets, evidencing the H_2O selective permeance on GO membrane during pervaporation separation (P. Saidi et al., *J. Phys. Chem. C* 124 (2020) 26864–26873). However, in this study, we confirmed higher adsorption amounts of H_2O over D_2O on GO for the entire range of P/P_0 by single-component adsorption isotherm measurements (see Fig. 4(a)) and mixed-vapor adsorption measurements (Fig. S2(b)). Thermogravimetric (TG) measurements and differential thermal analysis (DTA) of GO adsorbing water (see Fig. S14 and Fig. S15) indicated no clear difference in the desorption temperatures of physisorbed H_2O and D_2O. The number of strongly adsorbed D_2O molecules must be considerably less than that of the total water adsorbed in a realistic GO structure. This is because the adsorption sites for strongly adsorbed D_2O must be limited to only a part of the GO surface as compared with the adsorption sites for H_2O.

Under pervaporation separation measurements, lighter H_2O can permeate faster than D_2O through the GO membrane, resulting in higher H_2O content in the permeates, as reported by Saidi et al. Permeation is associated with the non-equilibrium-adsorbed states of H_2O and D_2O, which, in our experiment, were different from the adsorption states of H_2O and D_2O under equilibrium. We cannot discuss the relationship between the permeation and adsorption of H_2O and D_2O onto GO using the present adsorption data. Thus, this difference must be elucidated in another study.

3. Supplemental figures and tables

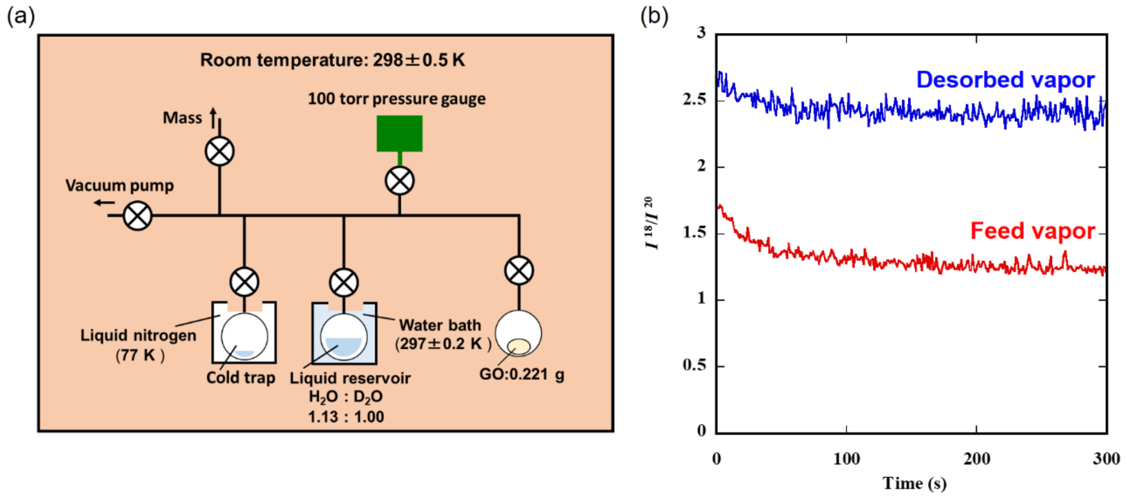

Figure. S2| H_2O selective adsorption experiment of mixed H_2O/D_2O vapors on GO. (a) Experimental setup for H_2O/D_2O mixed-vapor adsorption on GO using mass spectroscopy. **(b)** Time courses of the mass spectra for the feed vapor (red) and desorbed vapor (blue) after mixed-vapor adsorption on GO at 298 K and $P/P_0 = 0.94$ for 1 d. Here, the relative vapor pressure is for pure H_2O. The mass spectral intensity ratios for m/z = 18 and 20 are shown.

Table S1. Amounts of water isotopes adsorbed on GO in single-component and H_2O/D_2O mixed-vapor adsorption at $P/P_0 = 0.94$

	Mixed vapor adsorption	Single component adsorption
H_2O (mmol/g)	8.36	14.1
D_2O (mmol/g)	2.91	10.9
HDO (mmol/g)	9.68	-
H/D	1.70	1.29

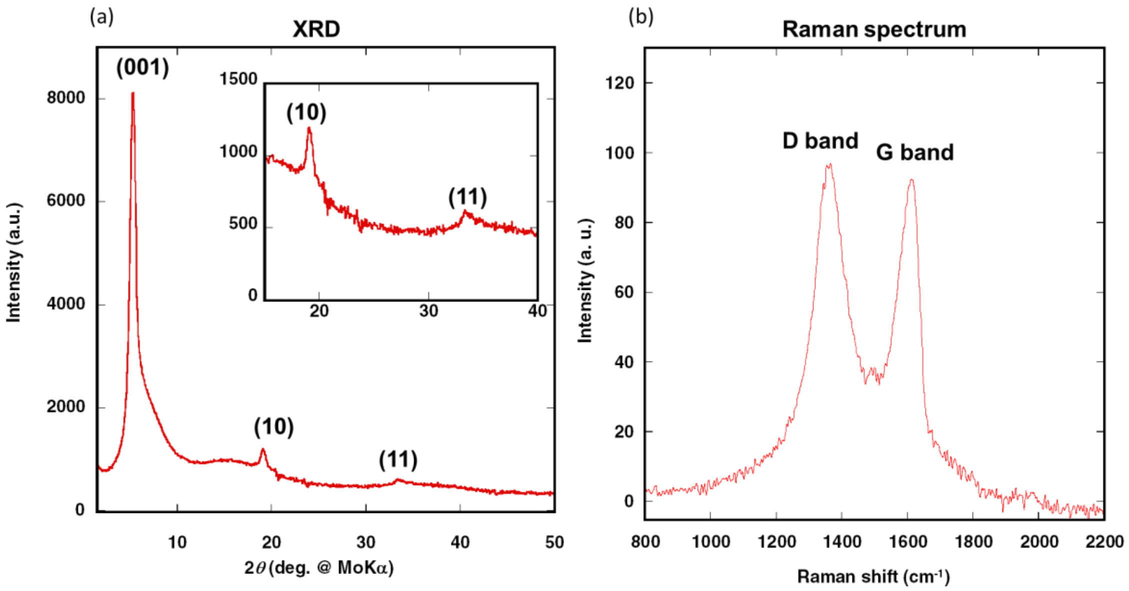

Figure. S3| (a)XRD and (b)Raman spectrum of GO.

Table S2. D and G-bands of Raman spectrum for GO

D band (cm^{-1})	G band (cm^{-1})	I_D/I_G	L_a (nm)
1360	1613	1.72	12

Table S3. The *d*-spacing and crystalline size of GO obtained from XRD

	2θ (degree)	*d* (nm)	*L* (nm)
(001)	5.65	0.72	4.7
(10)	19.1	0.21	11.0
(11)	33.3	0.12	6.5

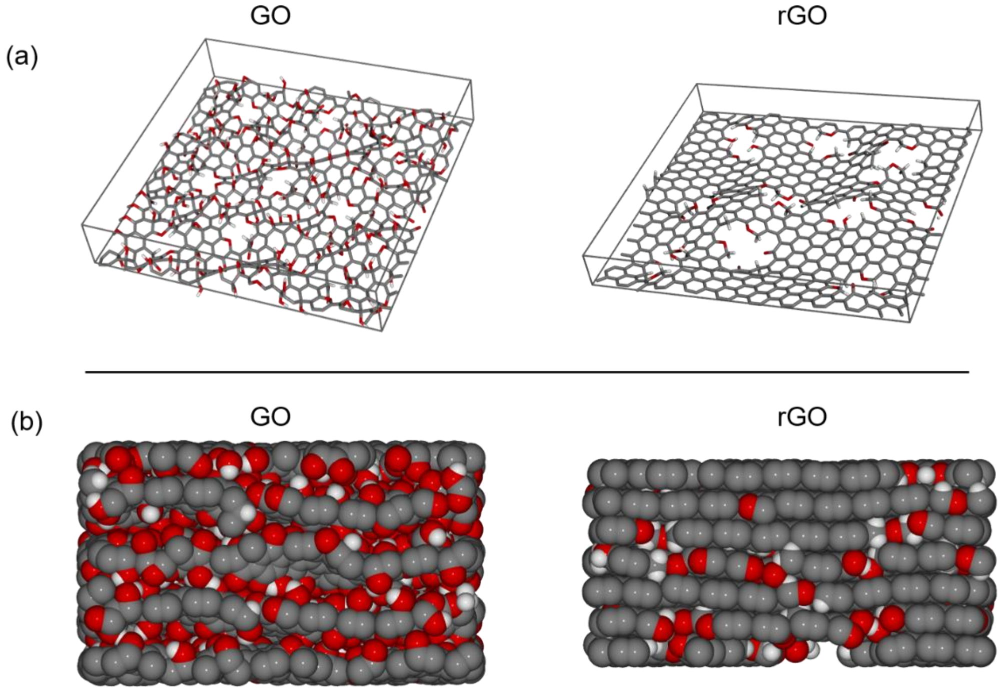

Figure. S4| (a) Snapshots of the sheet structure of an ultra-large GO layer and rGO layer after equilibrium on MD simulation. **(b)** Snapshots of the layered structures of each model after equilibrium on MD simulation. Here, we constructed the ultra-large GO and rGO layer models using the XPS data. We used the carbon atomic percentages for C-C/C=C, C-OH/C-O-C and C=O are 37, 44 and 19% in the GO sheet structure. The rGO model was constructed by removing oxygen functional groups from the model of simple stack ultra-large GO layer structure. The atomic percentage of carbons for C-C/C=C, C-OH/C-O-C and C=O are 89.1, 7.1 and 3.8% in the rGO sheet structure.

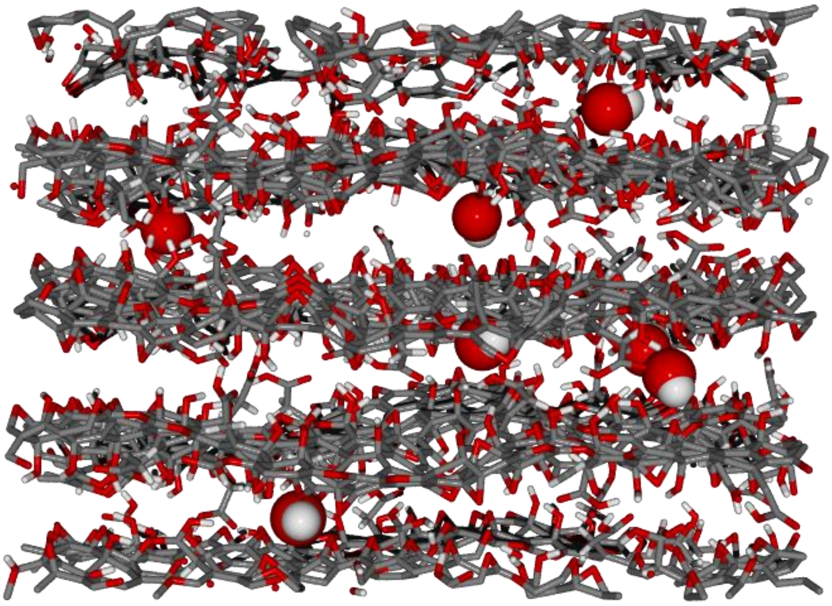

Figure. S5| Snapshot of reactions for functional groups and H_2O molecules in our MD simulation after 100ps calculation. Here, the products of water or hydroxyl groups are only shown as CPK models and the physisorbed H_2O molecules are omitted for simplicity.

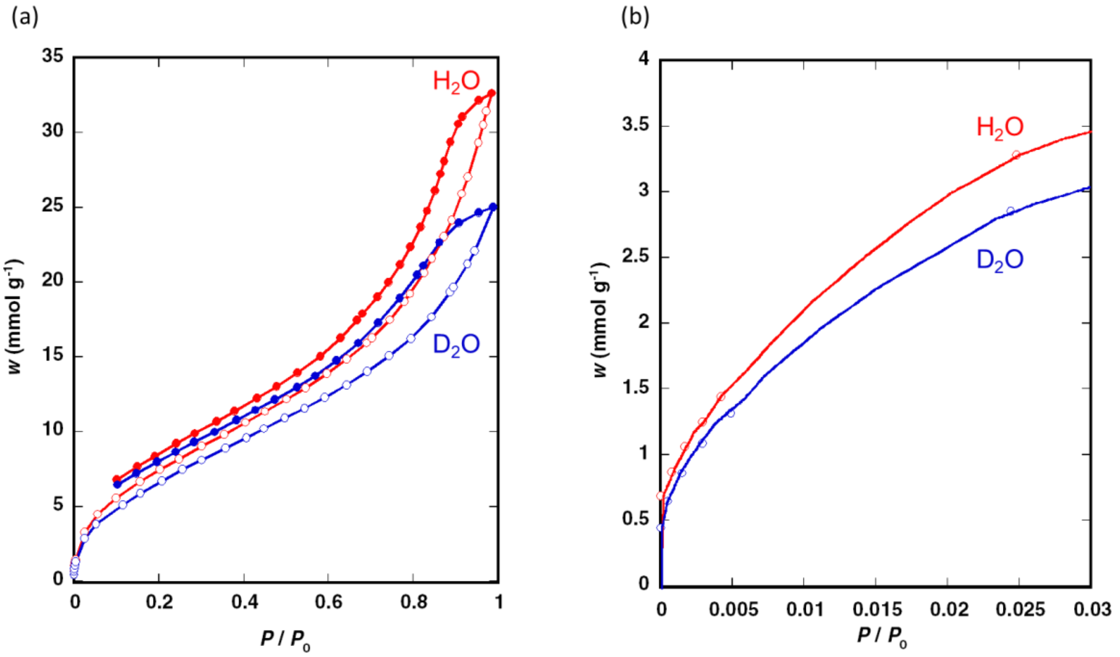

Figure. S6| (a) Single component adsorption isotherms of H_2O and D_2O on GO at 298 K. The vertical axis is expressed with the unit of mmol g^{-1}. **(b)** Magnified figures of the isotherms at low $P/P_0 < 0.03$.

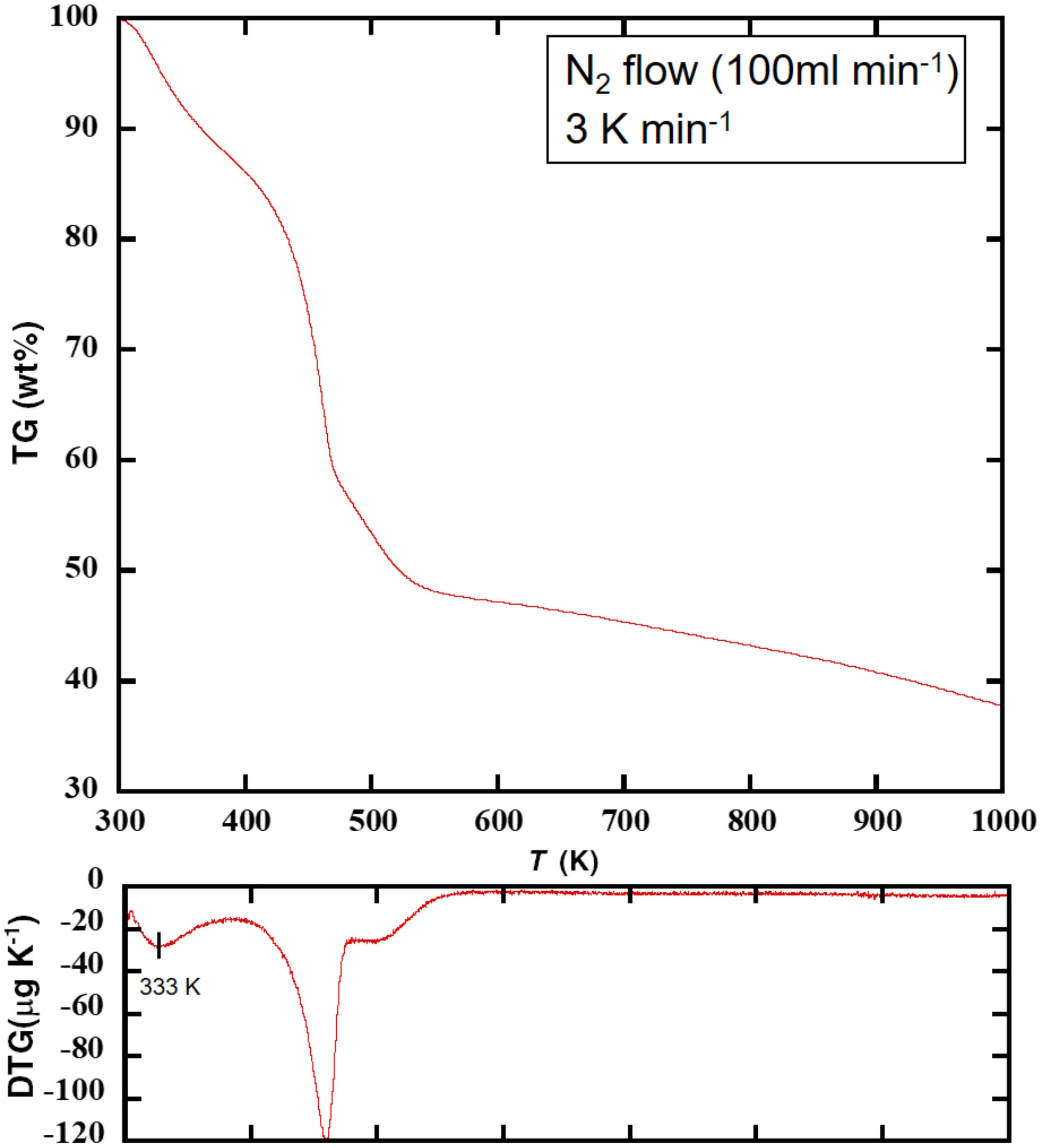

Figure. S7| Thermogravimetry (TG) and Derivative Thermogravimetry (DTG) for GO.

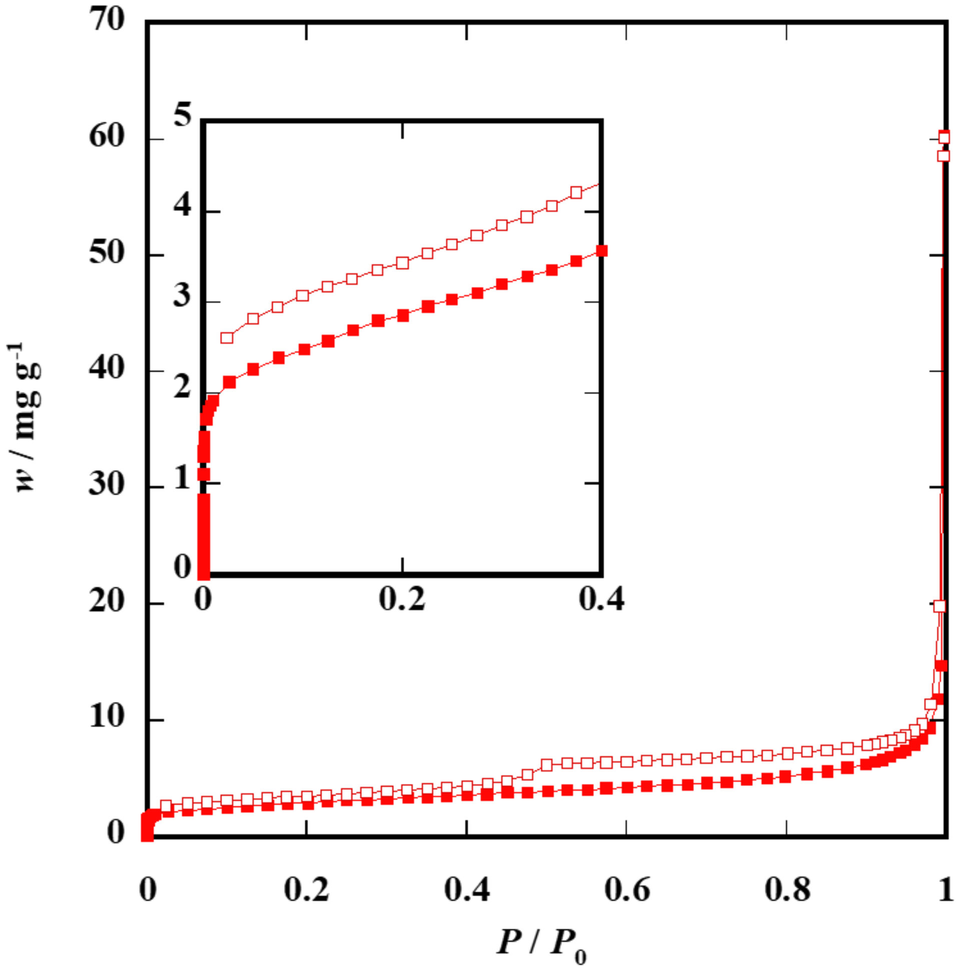

Figure. S8| Nitrogen adsorption isotherm on GO at 77 K.
(■ : Adsorption, □: Desorption)

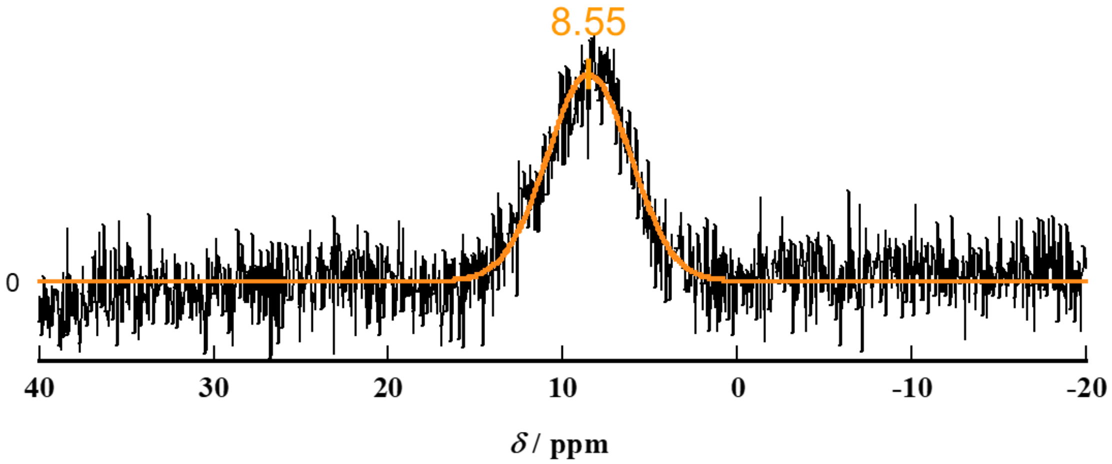

Figure. S9| *Ex-situ* ^{1}H-NMR spectra for H_2O adsorbed on GO at $P/P_0 = 0.1$.

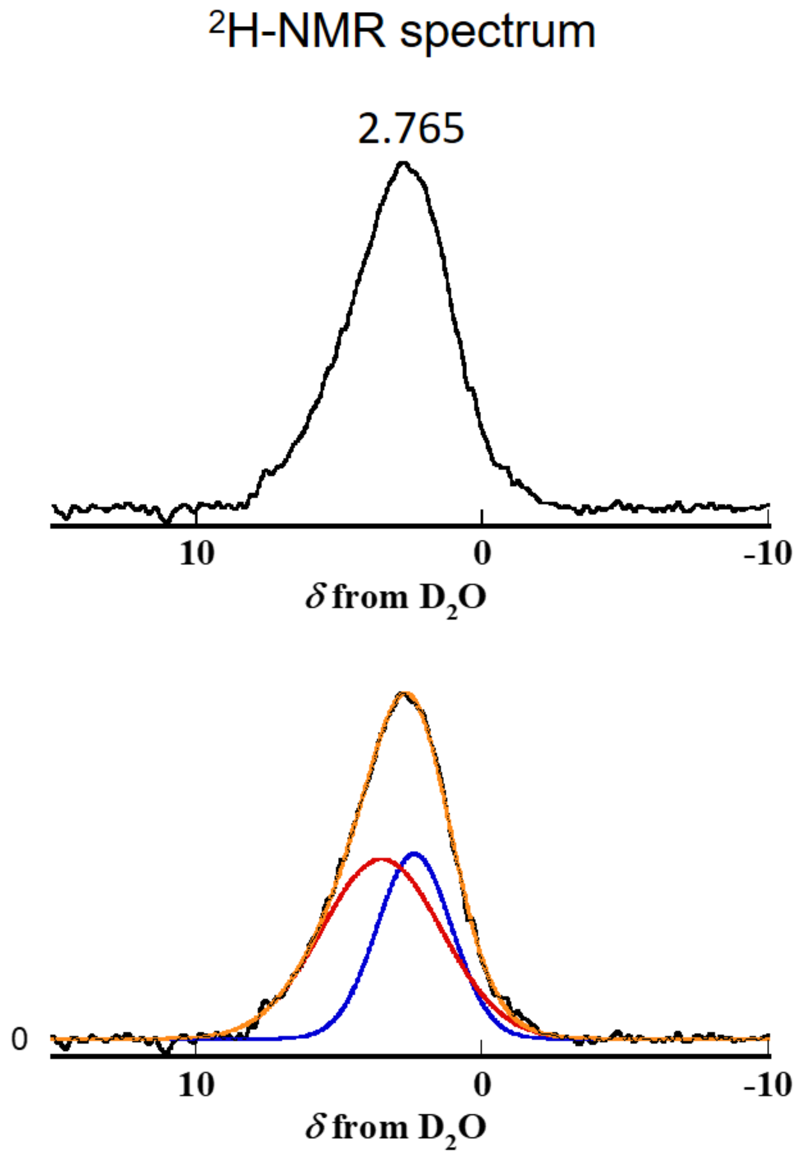

Figure. S10| *Ex-situ* ^{2}H-NMR spectra for H_2O adsorbed on GO at P/P_0 = 0.9 (top) and the deconvoluted result with two Gaussian functions (bottom).

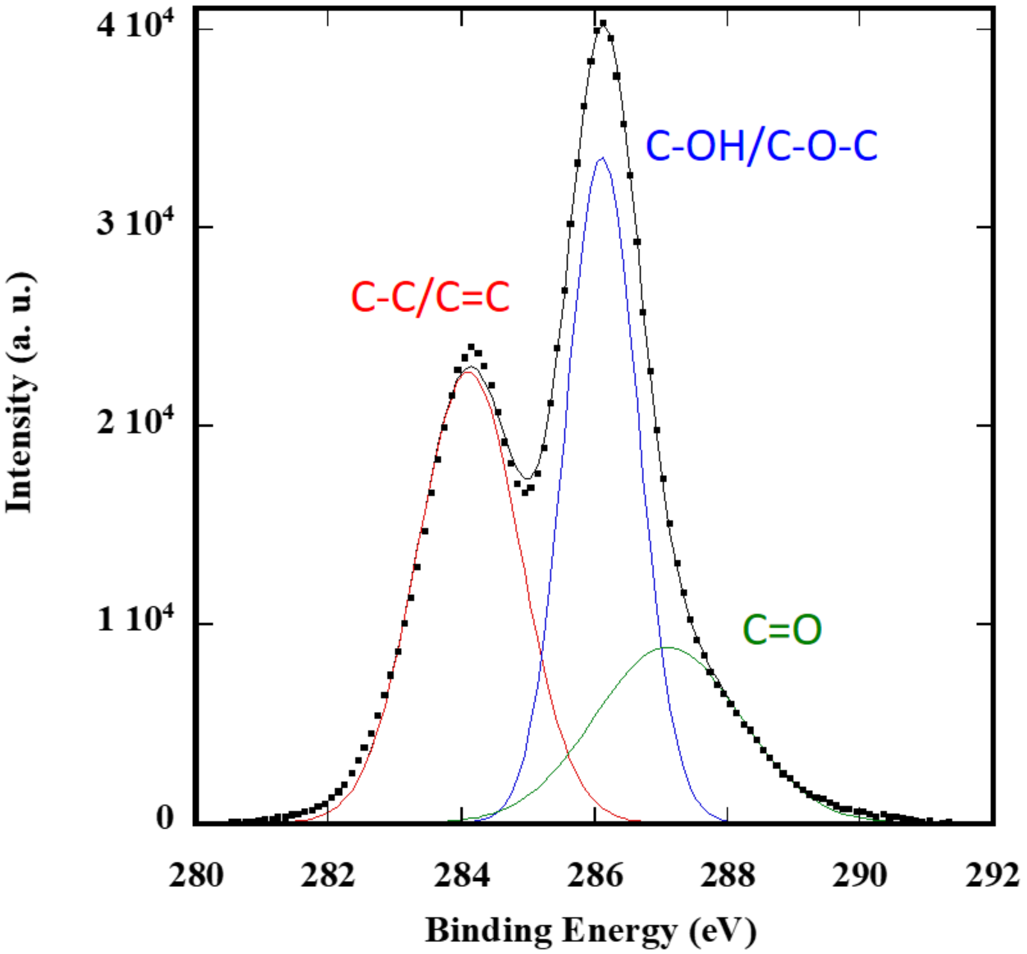

C1s	GOB1-C1s	
**Funct. group type**	position / eV	Atomic %
C-C/C=C	284.1	37
C-OH/C-O-C	286.1	44
C=O	287.3	19

Figure. S11| XPS narrow scan for C1s of GO.

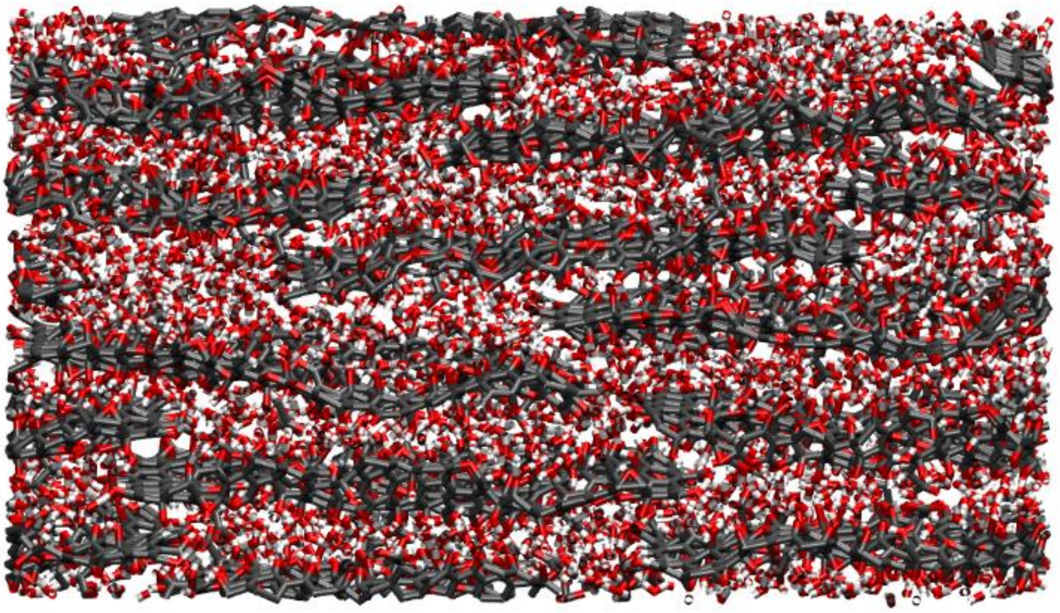

Figure. S12| Snapshot of partially staggered GO structure upon H_2O adsorption without compression. The interlayer spacing between GO sheets is 0.85 nm.

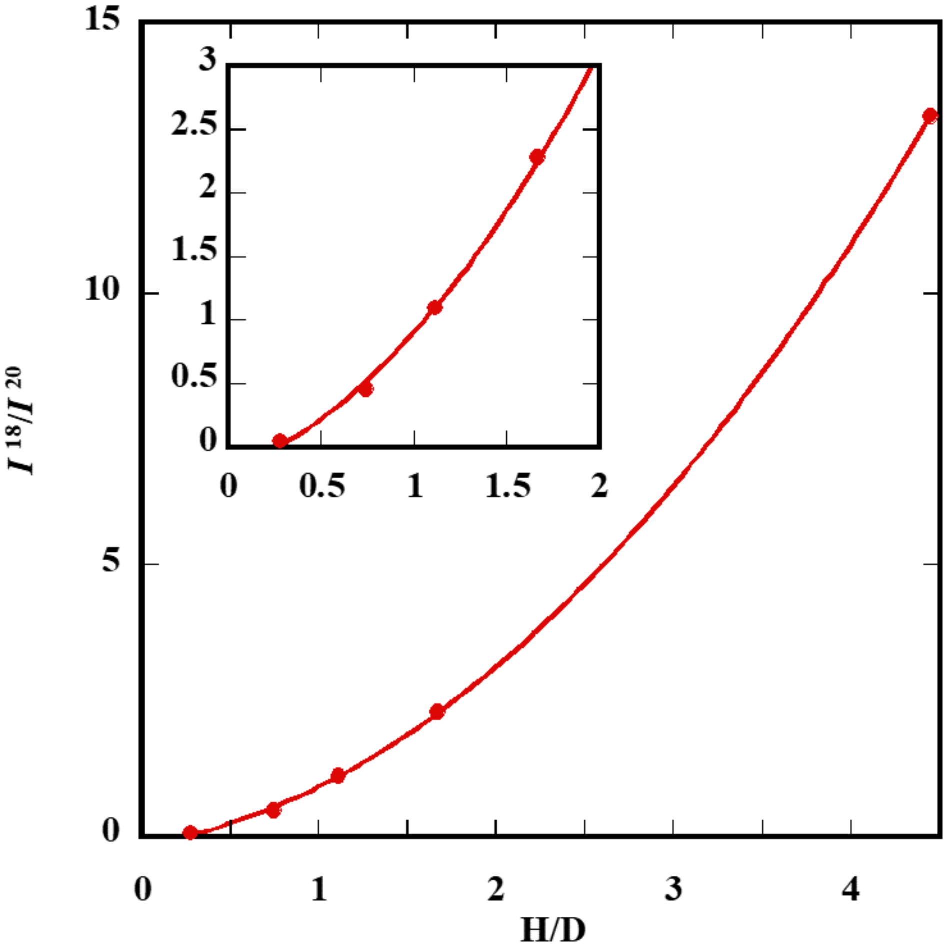

Figure. S13| Relationship between the H/D composition of the H_2O and D_2O vapor mixtures and the observed m/z = 18 and 20 intensity ratios (I^{18}/I^{20}) of the mixed vapors.

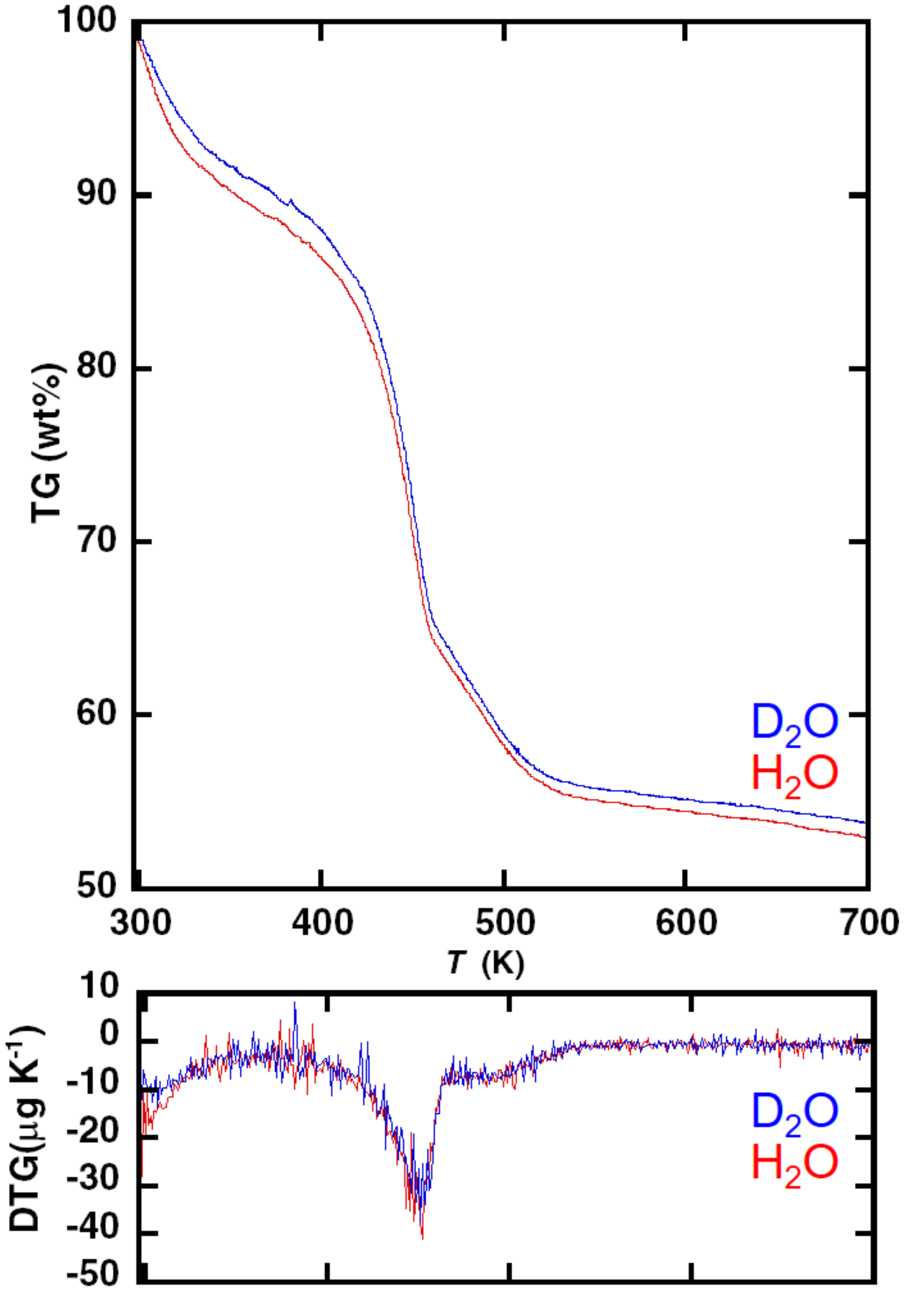

Figure. S14| TG profiles for GO adsorbing H_2O (red) and D_2O (blue) and their derivatives (DTG) conducted under N_2 flow at 250 mL min^{-1}. The heating rate was 1 K min^{-1}.

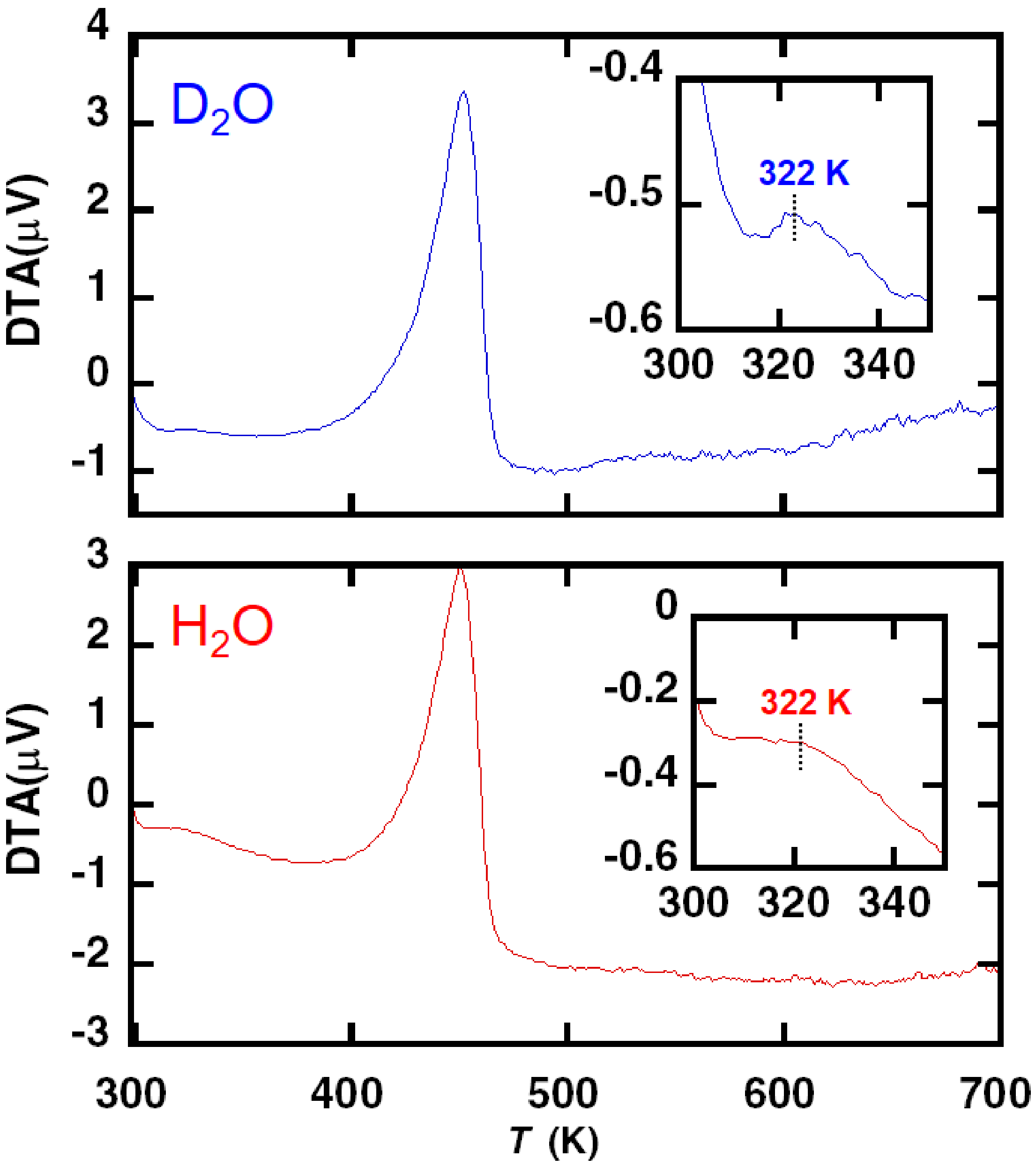

Figure. S15| DTA profiles for GO adsorbing H_2O (red) and D_2O (blue) conducted under N_2 flow at 250 mL min^{-1}. The heating rate was 1 K min^{-1}. (Insets) Magnified figures of the DTA profiles around 330 K< T < 350 K.

Table S4. Weights of GO samples before and after adsorption and desorption of H_2O and D_2O

	Before adsorption[1)] (g)	After adsorption[2)] (g)	After desorption[3)] (g)	Ratio of weights after desorption versus before adsorption
H_2O	0.101	0.126	0.102	1.01
D_2O	0.100	0.130	0.100	1.00

1) GO was pretreated at 333 K for 2 h under a vacuum (< 0.1 Pa) before the weight measurement.

2) Water vapor was adsorbed on GO at 295 K and P/P_0 = 0.9 for 1 d.

3) Water adsorbed on GO was desorbed under at 333 K and < 0.1 Pa for 2 h.

The following measurements were conducted to confirm that the H_2O and D_2O that were adsorbed onto GO were sufficiently desorbed under the desorption conditions of heating at 333 K *in vacuo* for 2 h. The weight[1)] of the GO sample prior to water adsorption was determined by preheating it at 333 K *in vacuo* for 2 h. The pretreated GO sample was placed in a desiccator containing saturated KCl H_2O or D_2O solutions for 1 d at 295 K to reach adsorption equilibrium. Subsequently, the weight[2)] of the GO sample with adsorbed H_2O or D_2O at the equilibrium adsorption amount was measured. Finally, the GO sample with adsorbed H_2O or D_2O was heated at 333 K *in vacuo* for 2 h to determine the weight[3)] of the GO sample following desorption. The determined weights are listed in Table S4.

The results presented in Table S4 ensure that adsorbed H_2O and D_2O are both thoroughly desorbed under the desorption conditions of heating at 333 K *in vacuo* for 2 h. The desorption conditions were used under mass spectroscopy to determine the adsorbed amounts of H_2O and D_2O in the mixed-vapor adsorption experiment. However, the weight measurement of GO with adsorbed water in atmosphere showed a slight error because of the inevitable desorption of water, which was unlike that in the single-vapor adsorption isotherm measurements.

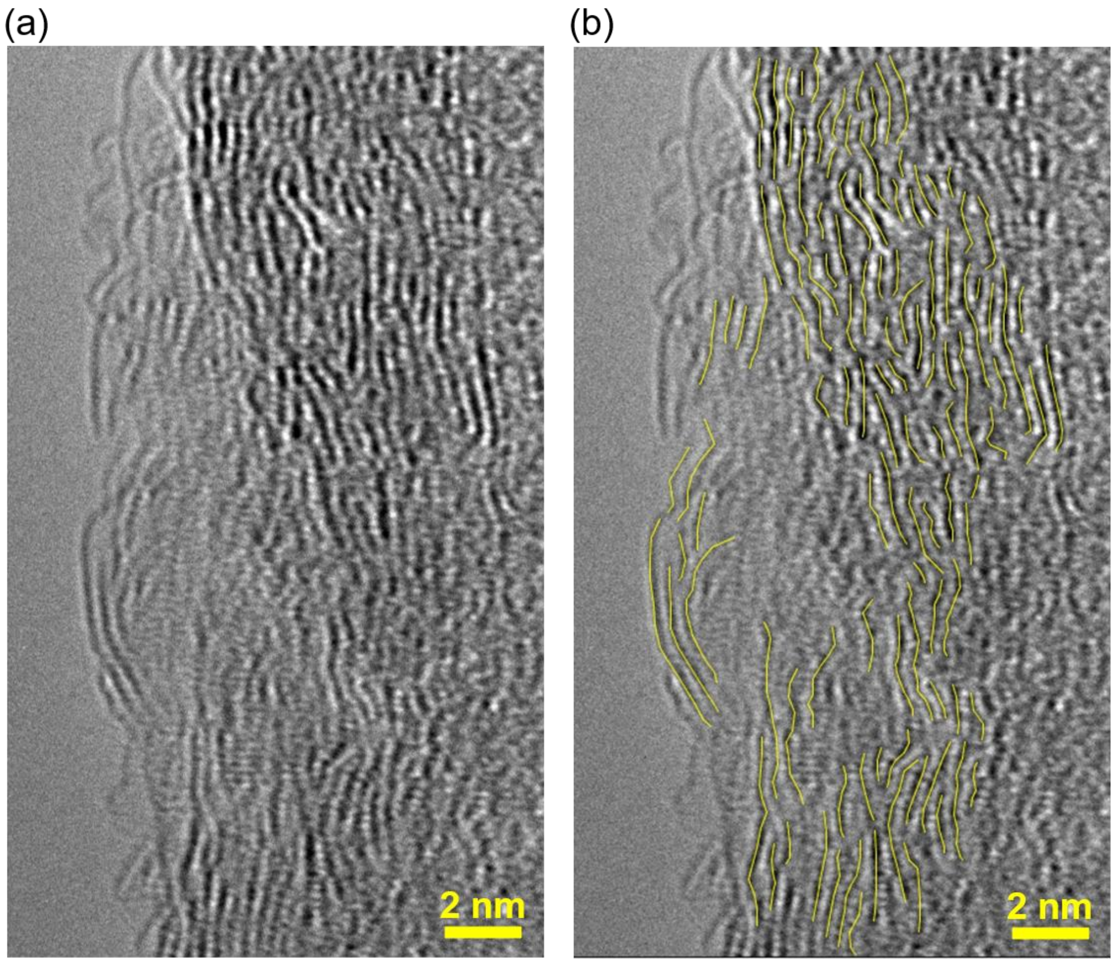

Figure. S16| (a)High-resolution transmission electron microscope (TEM) images of the staggered structures in Graphene oxide (GO). (b) The lateral sizes of GO sheets in the image of (a) are highlighted with yellow lines.
