# Supplementary material for: Staggered structural dynamic-mediated selective adsorption of H2O/D2O on flexible graphene oxide nanosheets
Source: Nat Commun. 2024 Apr 27;15:3585. doi: 10.1038/s41467-024-47838-9 (PMC11055881; doi:10.1038/s41467-024-47838-9)
Supplement: Supplementary file 2 — Description of Additional Supplementary Files [file 41467_2024_47838_MOESM2_ESM.pdf]

Description of Additional Supplementary Files

File name: Supplementary Data 1.dat
Description: Initial configuration of the MD simulation of simply stacked GO without H_2O molecules as shown in Figure 1(a).

File name: Supplementary Data 2.dat
Description: Final configuration of the MD simulation of simply stacked GO without H_2O molecules as shown in Figure 1(a).

File name: Supplementary Data 3.dat
Description: Initial configuration of the MD simulation of simply stacked GO with H_2O molecules as shown in Figure 1(a).

File name: Supplementary Data 4.dat
Description: Final configuration of the MD simulation of simply stacked GO with H_2O molecules as shown in Figure 1(a).

File name: Supplementary Data 5.dat
Description: Initial configuration of the MD simulation of staggered stacking GO without H_2O molecules as shown in Figure 1(b).

File name: Supplementary Data 6.dat
Description: Final configuration of the MD simulation of staggered stacking GO without H_2O molecules as shown in Figure 1(b).

File name: Supplementary Data 7.dat
Description: Initial configuration of the MD simulation of staggered stacking GO with H_2O molecules as shown in Figure 1(b).

File name: Supplementary Data 8.dat
Description: Final configuration of the MD simulation of staggered stacking GO with H_2O molecules as shown in Figure 1(b).

File name: Supplementary Data 9.dat
Description: Initial configuration of the MD simulation of simply stacked rGO without H_2O molecules as shown in Figure S4(b).

File name: Supplementary Data 10.dat
Description: Final configuration of the MD simulation of simply stacked rGO without H_2O molecules as shown in Figure S4(b).

File name: Supplementary Data 11.xyz
Description: The configuration of deformed functional groups and H_2O molecules in our MD simulation for the simply stacked GO with H_2O molecules as shown in Figure S5(b). Here, the physisorbed H_2O molecules are omitted

File name: Supplementary Data 12.dat
Description: Initial configuration of the MD simulation of staggered stacking GO adsorbing H_2O molecules without compression as shown in Figure S12.

File name: Supplementary Data 13.dat
Description: Final configuration of the MD simulation of staggered stacking GO adsorbing H_2O molecules without compression as shown in Figure S12.
